# Extracting cancer concepts from clinical notes using natural language processing: a systematic review

**DOI:** 10.1186/s12859-023-05480-0

**Published:** 2023-10-29

**Authors:** Maryam Gholipour, Reza Khajouei, Parastoo Amiri, Sadrieh Hajesmaeel Gohari, Leila Ahmadian

**Affiliations:** 1 Student Research Committee, Kerman University of Medical Sciences, Kerman, Iran; 2 Department of Health Information Sciences, Faculty of Management and Medical Information Sciences, Kerman University of Medical Sciences, Kerman, Iran; 3 Medical Informatics Research Center, Institute for Futures Studies in Health, Kerman University of Medical Sciences, Kerman, Iran

**Keywords:** Neoplasms, Natural language processing, NLP, Machine learning, Terminology, Information system, Systematic review

## Abstract

**Background:**

Extracting information from free texts using natural language processing (NLP) can save time and reduce the hassle of manually extracting large quantities of data from incredibly complex clinical notes of cancer patients. This study aimed to systematically review studies that used NLP methods to identify cancer concepts from clinical notes automatically.

**Methods:**

PubMed, Scopus, Web of Science, and Embase were searched for English language papers using a combination of the terms concerning “Cancer”, “NLP”, “Coding”, and “Registries” until June 29, 2021. Two reviewers independently assessed the eligibility of papers for inclusion in the review.

**Results:**

Most of the software programs used for concept extraction reported were developed by the researchers (*n* = 7). Rule-based algorithms were the most frequently used algorithms for developing these programs. In most articles, the criteria of accuracy (*n* = 14) and sensitivity (*n* = 12) were used to evaluate the algorithms. In addition, Systematized Nomenclature of Medicine-Clinical Terms (SNOMED-CT) and Unified Medical Language System (UMLS) were the most commonly used terminologies to identify concepts. Most studies focused on breast cancer (*n* = 4, 19%) and lung cancer (*n* = 4, 19%).

**Conclusion:**

The use of NLP for extracting the concepts and symptoms of cancer has increased in recent years. The rule-based algorithms are well-liked algorithms by developers. Due to these algorithms' high accuracy and sensitivity in identifying and extracting cancer concepts, we suggested that future studies use these algorithms to extract the concepts of other diseases as well.

## Background

One of the significant public health concerns is cancer. According to the World Health Organization (WHO) report in 2019, this disease is the leading cause of death worldwide [1]. GLOBOCAN (The Global Cancer Observatory) estimated [2] about 10 million deaths from cancer in 2020 (i.e., one in every six patients with cancer) [3]. The global cancer-related deaths are predicted to be around 13 million by 2030 [4]. Due to the growing incidence of cancer, researchers use various methods to combat this disease. Artificial intelligence (AI) is one of the methods that has been used to diagnose cancer [5–9] and predict its risk [10], relapse [11], and symptoms [11–13]. AI can provide a safe, fast, and efficient way to manage such diseases.

Natural language processing (NLP) is a branch of AI that addresses the interpretation and comprehension of texts using a set of algorithms [13–15]. NLP is the key to obtaining structured information from unstructured clinical texts [16]. Today, large amounts of clinical information are recorded and stored as narrative text in electronic systems. Retrieving and using this information can facilitate the diagnosis, treatment, and prediction of diseases. So far, NLP has been widely used in medical and health research, e.g., for identifying care coordination terms in nursing records [17], identifying medical concepts from radiology reports [18], extracting complications from problem lists [19], and determining disease status in discharge summaries [20]. For example, Si et al. [21] proposed a framework-based NLP method for extracting cancer-related information with a two-step strategy including bidirectional long short-term memory and conditional random field. Other studies extracted tumor-related information, such as location and size, using the NLP method [22, 23]. Kehl et al. [24] reported that the neural network-based NLP method could extract significant data from oncologists' notes.

Due to the unique characteristics of clinical texts, such as poor structure, use of specific vocabulary, and abbreviations [24] that make the use of NLP challenging, understanding the new developments of NLP in clinical research is essential. Despite the various studies that have been done on the application of NLP in medicine, there are limited systematic review studies summarizing its application. Previous systematic reviews mostly addressed the extraction of concepts from clinical texts such as radiology, laboratory, pathology, evaluation of postoperative surgical results, assessment of the application of NLP in the clinical practice of mental health, and development and adoption of NLP methods in open-text clinical notes related to chronic diseases [16, 25–29]. Casey et al. [25] investigated the use of NLP algorithms that were used in various studies to analyze radiology reports. In this study, besides determining the NLP algorithms, they focused on the purpose of using these algorithms for analyzing the reports and reported the following main applications: disease information and classification, language discovery and knowledge structure, quality and compliance, and cohort and epidemiology. In their systematic review, Pons et al. [15] also investigated NLP methodologies used on radiology reports and described the application and the purpose of using NLP, the tools used, and the performance results. Concerning the application of NLP in cancer, Santos conducted a study on NLP algorithms and extracted information regarding various models applied in different studies and their performances [24]. Based on the results of a systematic review of the application of NLP models to evaluate postoperative surgical outcomes, the most common outcome was postoperative complications. These complications can be identified more reliably using NLP models compared to traditional non-NLP alternatives. Glaz et al. [27] evaluated studies that used machine learning and NLP techniques in the field of mental health and also the potential application of these methods in mental clinical practice. The main objectives were to extract terms related to symptoms, classify the severity of illness, compare therapy effectiveness, and provide psychopathological clues. Sheikhalishahi et al. [28] carried out a comprehensive overview of the development and uptake of NLP methods in open-text clinical notes related to chronic diseases, including an examination of the challenges of using NLP methods to extract terms from clinical narratives. The results of this study showed a trend indicating that most studies focused on cardiovascular diseases, ،while endocrine and metabolic diseases were the least researched topics. This trend may occur because clinical records related to metabolic diseases are more structured than those related to cardiovascular diseases.

Given the increasing incidence of cancer, as well as the recent advancements of NLP techniques to assist with the parsing and analysis of cancer-specific medical literature, a new systematic review in this area can help researchers and professionals gain a deeper understanding of this field and identify new techniques in cancer research to support and promote cancer research. To our knowledge, all existing systematic reviews addressed extracting NLP algorithms, and none of them specifically focused on extracting cancer concepts and the terminologies applied to detect the information regarding different types of cancer. Therefore, in this study, we systematically reviewed the studies on extracting cancer concepts to determine which NLP methods have been applied to automatically identify cancer concepts in clinical notes, which terminologies are used to code cancer concepts, and what types of cancers are identified. The results of this study can help researchers identify the existing NLP methods and proper terminological systems in this field.

## Method

This systematic review was performed using the guidelines of the Preferred Reporting Items for Systematic Reviews and Meta-Analyses (PRISMA) [30]. PRISMA is a guideline that helps researchers to format their reviews and demonstrate the extent of the quality of their reviews. Also, the present study used wordcloud to pinpoint which variables need to be highlighted.

### Information resources and searches

The PubMed, Scopus, Web of Science, and Embase databases were searched for relevant literature until June 29, 2021. A list of terms, keywords, and their synonyms were identified and categorized into four groups: "Cancer", "NLP", "Coding", and "Registries". We used the "OR" operator to combine the expressions within groups 1, 2, 3, and 4 and the "AND" operator to combine the results of the four groups (Table 1 shows the keywords for each group).

**Table 1:** Groups of keywords used in the search strategy

Group 1	Automatic mapping OR Automatic coding OR Lexical mapping OR Text parsing OR Text mining OR **NLP** OR **Natural Language processing** OR Information extraction OR Text understanding OR Text analysis OR Concept extraction OR Concept mapping OR Narrative parsing OR Automatic annotation
Group 2	Coding OR **Terminology** OR Thesaurus OR **Vocabulary** OR Glossary OR **Nomenclature** OR **Classification** OR Taxonomy OR Ontology OR Terminological system OR Nosology OR Lexicon
Group 3	Cancer OR **Neoplasms** OR Neoplasia OR Tumor OR Malignancy OR **Anatomical Pathological Condition** OR oncology OR Morphology OR **Cell differentiation** OR **Lymphoma** OR **Carcinoma** OR **Adenoma** OR **Sarcoma** OR **Fibroma** OR **Thymoma** OR **Leukemia** OR **Papilloma** OR **Pathology**
Group 4	**Registries** OR **Information System** OR **Database** OR **Surveillance system**

*MeSH terms are in bold

### Inclusion criteria

All articles included in the study were original research articles that sought to retrieve cancer-related terms or concepts in clinical texts. These articles used the NLP technique to retrieve cancer-related concepts.

### Exclusion criteria

Articles that used the NLP technique to retrieve concepts related to other diseases were excluded from the study. Studies that used the NLP technique in the field of cancer but used this technique to extract tumor features, such as tumor size, color, and shape, were also excluded. In addition, articles that used the NLP technique to diagnose cancer based on the patient's clinical findings were not included in the study. For example, articles that aimed to diagnose cancer based on the results of biomarker tests and measurements in the patient's body and the symptoms were not eligible for inclusion in the study. Furthermore, all review articles, conferences, and articles that retrieved cancer concepts from animal medical records were also excluded.

### Article selection

**Fig. 1:**
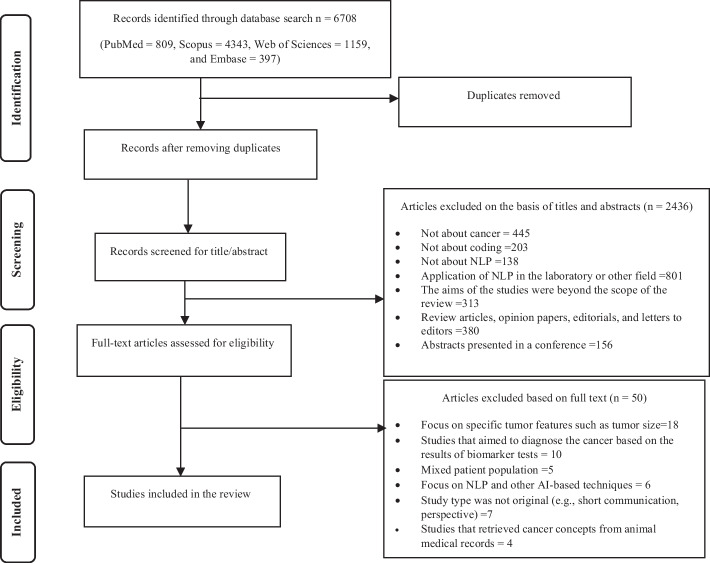
The PRISMA diagram of study selection

Articles retrieved from databases were first entered into EndNote version X10. After eliminating duplicate studies, two authors (M.Gh and P.A) independently reviewed the titles and abstracts of the retrieved articles. Figure 1 shows the PRISMA diagram for the inclusion and exclusion of articles in the study. After deleting irrelevant articles, the full text of the related articles was independently reviewed by three authors (S.Hg, M.Gh, and P.A). Disagreements among the reviewers were resolved by consensus in a meeting with another author (L.A).

### Data collection process

A data extraction form was developed by the researchers. The validity of this form was confirmed by three medical informatics specialists and a health information management specialist. The form included the following headings: Authors, Year of publication, Setting, System, System module, Objective, Cancer type, Outcome, Data standard exchange, Terminological systems, NLP type, and Algorithm.

## Results

This study was a systematic review that aimed to review articles that extracted cancer concepts using NLP. In total, 6708 papers were initially retrieved. After removing duplicates, 2503 articles remained for further review. Subsequently, the titles and abstracts of the remaining articles were screened, and inclusion and exclusion criteria were applied. After applying exclusion criteria, a total of 2436 articles were excluded, and 67 studies were deemed relevant. The full texts of these articles were reviewed, and finally, 17 articles were selected, and their information was extracted (Fig. 1).

## General characteristics of the included articles

**Fig. 2:**
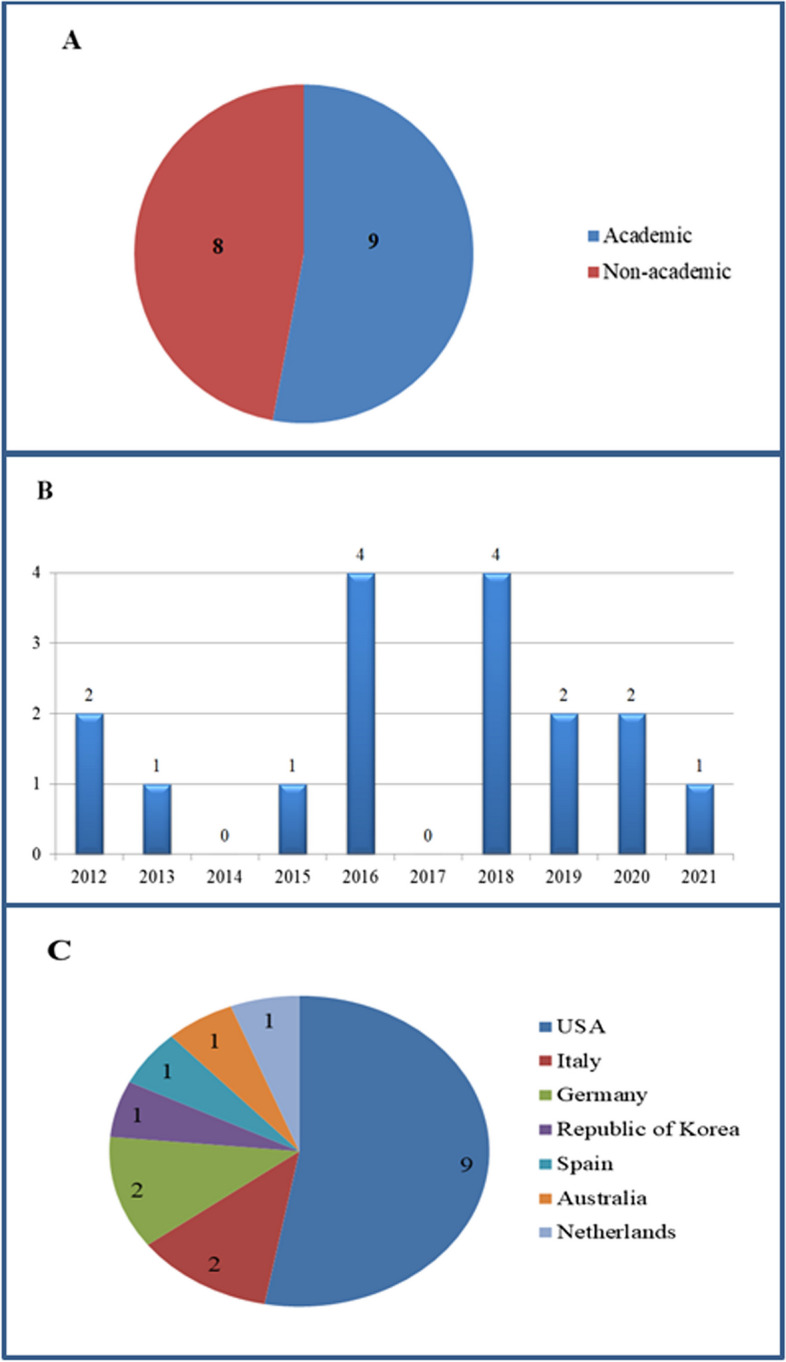
General characteristics of included studies: **A** number of academic and non-academic articles; **B** number of articles per year; **C** number of articles per country

The publication dates of the retrieved articles were between 2012 and 2021 (Fig. 2). Most articles were published between 2016 and 2020. Out of 17 articles included in the present study, 10 were conducted in non-academic settings (*n* = 10, 58%).

## Aims of the included articles

The aims of these 17 articles were evaluated after reviewing the full text of the articles (Table 2). The study aims were divided into four general categories: “extraction of cancer concepts” (*n* = 12), “comparison of the results retrieved by NLP algorithms and manual coding” (*n* = 3), “comparison of different NLP algorithms in terms of their ability to extract cancer concepts” (*n* = 1), and “extraction of cancer concepts and coding” (*n* = 1).

**Table 2:** The aims of the included articles

Aim type	References
Extraction of cancer concepts	[31–42]
Comparison of the retrieval results of NLP algorithms with manual coding	[43–45]
Comparison of different NLP algorithms in terms of their ability to extract cancer concepts	[45, 46]
Extraction of cancer concepts and coding	[47]

## Specific characteristics of the included articles

System, Module and Database characteristics of the included articles are shown in Table 3.

**Table 3:** System, module, and database characteristics of the included articles

Author	System	Module	Database
Hammami et al. [36]	–	Pathology reports	Oracle Data Warehouse of Fondazione IRCCS “Institute nazionale dei tumori” (Istituto Nazionale dei Tumori)
Ryu et al. [37]	EHR	Pathology reports	–
Oliveira et al. [41]	–	Cervical and anal pathology reports	Clinical pathology laboratory information system
Becker et al. [48]	EHR	Clinical note	–
Wang et al. [40]	Mayo clinic EHR	Clinical notes and pathology reports	–
Kumar et al. [33]	–	Pathology reports	The Dartmouth-
			Hitchcock Medical Center (DHMC)
Wadia et al. [43]	–	Radiology reports (Ct)	Clinical text analysis
		Chest CT reports	
Bustos et al. [38]	–	Public registry	Free dataset
Faina Linkov et al.[47]	–	UPMC registry	–
Sada et al. [34]	–	Liver pathology reports, abdominal CT, and abdominal MRI reports	Veterans Affairs administrative data
Nguyen et al. [44]	–		
Hoogendoorna et al. [45]	–	–	Primary care dataset originating from a network of general practitioners (GPs) centered around the Utrecht University Medical Center
Löpprich et al. [39]	–	Clinical report	Multiple myeloma research database
Mehrabi et al. [32]	–	–	Indiana University (IU) dataset
			Mayo Clinic dataset
Sippo et al. [42]	EMR	Breast imaging reports	–
Segagni et al. [31]	HIS and biobank	FSM pathology unit hospital biobank	FSM pathology unit database
Strauss et al. [46]	EMR	Pathology report	–

The Algorithm performance characteristics of the included articles are shown in Table 3. -: Indicates that the information are not reported in the included studies

### System

The data analyzed in the included articles were extracted from various resources such as databases, registers, and health information systems. Data from multiple databases were examined in 10 out of the 17 articles included in the present study. In two of these 10 articles, more than one database was used. In three articles, electronic health record (EHR) data were examined. In these articles, clinical notes, pathology reports, and surgery reports were analyzed. In two articles, the data were retrieved from the electronic medical records (EMR) system, and the reports analyzed in these systems were breast imaging and pathology reports. In one article, the cancer registry, the Surveillance, Epidemiology, and End Results (SEER) registry data, pathology reports, and radiology reports were examined.

### NLP type and algorithm performance

NLP type and Algorithm performance articles are shown in Table 4.

**Table 4:** NLP type and Algorithm performance characteristics of the included articles

Author	NLP type (algorithm)	Performance
Hammami et al. [36]	Developed by researchers (rule-based)	86.3% < = Sens < = 99.2%
		85.9% < = Prec < = 99.2%
		84.9% < = F1-score < = 99.2%
		98.10% < = Acc < = 99.2%
Ryu et al. [37]	Developed by researchers (rule-based)	Sens = 100%
		98.6 < = Prec < = 100
		99.3 < = F1-score < = 100
Oliveira et al. [41]	Developed by researchers Developed by researchers (rule-based + ML) + CLAMP software	–
Becker et al. [48]	Developed by researchers (semi-automated rule-based system)	Sens = 99.54%
		Prec = 97.95%
Wang et al. [40]	Developed by researchers (algorithms to generate final normalized concept names for each data element) + ML + Premade (Med Tagger)	0.982 < = Recall < = 1
		0.885 < = Prec < = 1
Kumar et al. [33]	Developed by researchers (semantic similarity measures and clustering methods) + cTAKES	*P *value < = 0.05
Wadia et al. [43]	Premade (cTAKES)	Sens = 77.3
		Spec = 72.5
		Prec = 88.4
		NPV = 54
Bustos et al. [38]	Developed by researchers	0.79 < = Sens < = 0.93
	((Fast Text. CNN, SVM, KNN))	0.79 < = Prec < = 0.93
		0.79 < = F1-score < = 0.93
Faina Linkov et al.[47]	Premade (TIES)	–
Sada et al. [34]	Premade (ARC = automated retrieval console)	0.94 < = Sens < = 0.96
		0.68 < = Spec < = 0.97
		0.75 < = PPV < = 0.96
Nguyen et al. [44]	Premade	Sens = 0.78
	( Medtex) + Developed by researchers (rule-based)	Prec = 0.83
		F1-score = 0.80
Hoogendoorna et al. [45]	Developed by researchers (rule-based)	0.870 < = Accu < = 0.831
Löpprich et al. [39]	Developed by researchers (SVM—MEC) + German framed clinical text	0.89 < = F1-score < = 0.92
Mehrabi et al. [32]	Developed by researchers (rule-based)	87.8 < = Prec < = 88.1
Sippo et al. [42]	Premade (BROK)	Sens = 100%
		96.6% < = Prec < = 100%
Segagni et al. [31]	Developed by researchers and premade	–
	(rule-based system for the onco-i2b2 + researcher-made algorithm	
Strauss et al. [46]	Premade (CoPathPlus) + Developed by researchers (Scent = rule-based)	Breast cancer
		0.74 > Sens > 1
		Spec = 0.99
		0. > 90 Prec > 0.94
		0.97 < NPV > 1
		Prostate cancer
		0.71 > Sens > 0.97
		0.9 > 8 Spec < 0.99
		0.88 > Prec > 0.97
		0.95 > NPV > 0.99

NLP type and Algorithm performance articles are shown in Table 4.

Accu: Accuracy, Sens: Sensitivity, Spec: Specificity, Prec: Precision, SVM: support vector machines, Scent: SAS-based coding, extraction, and nomenclature tool, ML: Machine Learning.

Extensive variations were observed in the software and algorithm evaluation methods used in the articles included in the present study. The reported precision in 14 articles was between 65 and 99% (*n* = 14, 82%), sensitivity in 12 articles was between 57 and 100% (*n* = 12, 70%), f1-score in 9 articles was between 45 and 99% (*n* = 9, 52%), specificity in 3 articles was between 72 and 99% (*n* = 3, 17%), and accuracy in 2 articles varied between 98 and 100% (*n* = 2, 11%).

### Terminological systems

Terminological System and Data standard exchange and Cancer type characteristics of the included articles in Table 5

**Table 5:** Terminological system and data standard exchange and cancer type characteristics of the included articles

Author	Terminological system	Data standard exchange	Cancer type
Hammami et al. [36]	ICD-O-M	–	All
Ryu et al. [37]	LOINC, ICD-O, SNOMED CT- OMOP- Local dictionary	–	Colon
Oliveira et al. [41]	–	–	Cervical and anal
Becker et al. [48]	UMLS	–	Colorectal
Wang et al. [40]	–	–	Lung
Kumar et al. [33]	SNOMED-CT – UMLS	–	Lung
Wadia et al. [43]	UMLS – SNOMED-CT, ICD-9, customized dictionary of additional terms added to SNOMED-CT	–	Lung
Bustos et al. [38]	–	XML	All
Faina Linkov et al.[47]	ICD_O, ICD_9		
ICD 10	NAACCR	Breast cancer	
Sada et al. [34]	ICD-9	–	Hepatocellular
Nguyen et al. [44]			All
Hoogendoorna et al. [45]	UMLS—SNOMED-CT	–	Colorectal
Löpprich et al. [39]	UMLS – open NLP- NP-channker – pos Tagger	–	All
Mehrabi et al. [32]	UMLS	HL7-CDA	Pancreatic
Sippo et al. [42]	–	Java	Breast
Segagni et al. [31]	SNOMED CT, malignant tumors (TNM) classifications	A web service that communicates with other cells through XML messages following REST standard	ALL
Strauss et al. [46]	SNOMED	–	Breast & Prostate

The Terminological system's characteristics of the included articles are shown in Table 5.

LOINC: Logical Observation Identifiers Names and Codes, ICD-O: International Classification of Diseases for Oncology, SNOMED CT: Systemized Nomenclature of Medicine – Clinical Terms, OMOP: Observational Medical Outcomes Partnership, CLAMP software: Clinical Language Annotation Modeling and Processing, UPMC: University of Pittsburgh Medical Center, TIES: Text Information Extraction System, NAACCR: North American Association of Central Cancer Registries, FSM: Fondazione Maugeri hospital, i2b2: Informatics for Integrating Biology and the Bedside, TNM: T describes the size of the tumor and any spread of cancer into nearby tissue; N describes the spread of cancer to nearby lymph nodes; and M describes metastasis, XML: an extensible markup language.

The most frequently used terminologies were UMLS and SNOMED-CT. In six of the 17 articles, the data exchange standard was used for data transfer; in two articles, the HL7 standard; in two articles, the XML standard; in two articles, the JAVA standard; and in one article, the CDA standard was employed.

### Cancer type

Of all the articles reviewed, 70% focused on a specific type of cancer, with breast cancer (*n* = 4, 19%) and lung cancer (*n* = 4, 19%) receiving the most attention.

## Results from wordcloud analysis

**Fig. 3:**
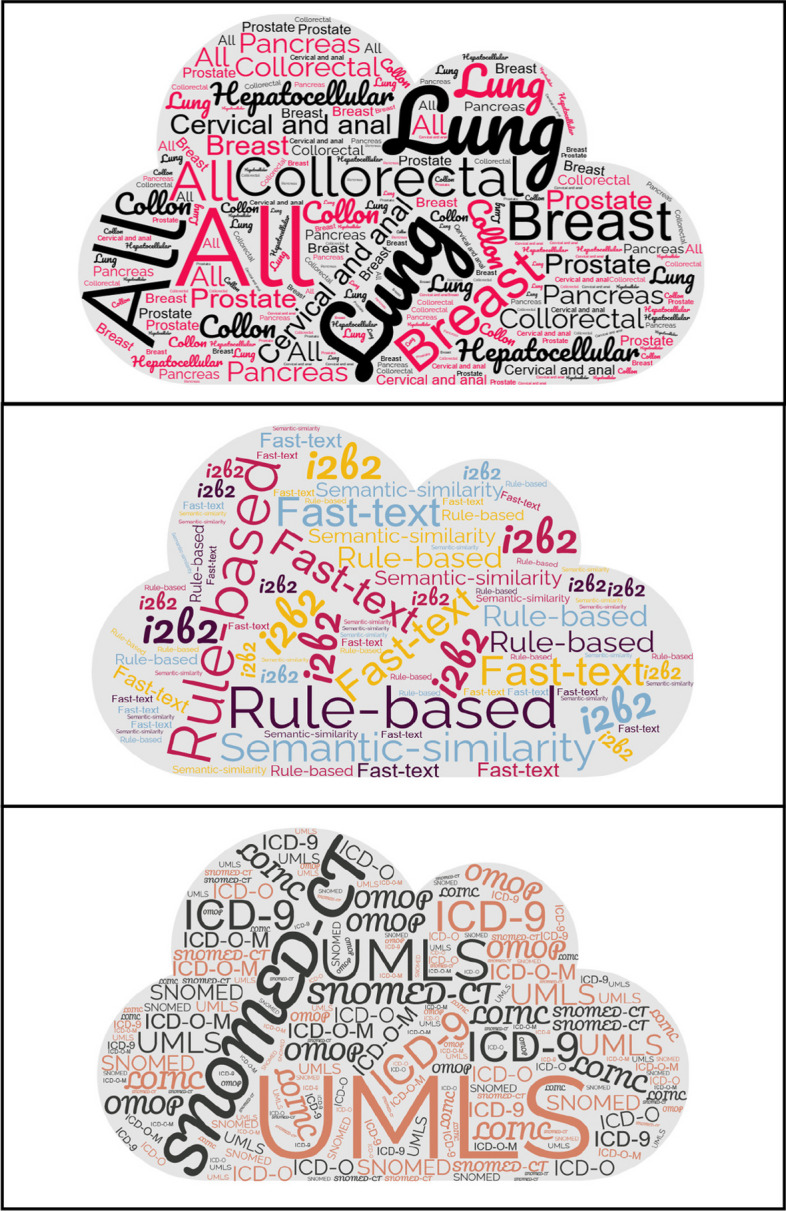
A wordcloud view of extracted three variables (cancer types, algorithms, terminologies)

The wordclouds of three variables (cancer types, algorithms, terminologies) are presented in Fig. 3. The wordclouds represents the most common terms used in the included articles. The more frequent a word, the bigger and more central its representation in the cloud.

## Discussion

This study aimed to review and synthesize the results of the articles focusing on concept retrieval concerning cancer using NLP software. The most commonly used terminologies in the articles included in this study were SNOMED, SNOMED-CT, and UMLS. Studies that evaluated only one or more specific types of cancer provided data on high-prevalence cancers such as breast, colon, and lung cancer. Moreover, the most frequently used algorithm in the software development of these studies was the rule-based algorithm. In recent years, the number of studies that used NLP to retrieve and extract concepts and words has increased (*n* = 70%), which confirms a growth in the use of NLP in medicine [1, [49]. With the development of health information systems, electronic information registration, and electronic preparation of medical reports, the volume of textual data recorded in these systems has increased. The rise in the diversity and volume of data prompted researchers to use various techniques to retrieve these texts.

NLP applications provide a significant advantage via automation. They effectively reduce or even eliminate the need for manual narrative reviews, which makes it possible to assess vast amounts of data quickly. As a consequence, previously impractical tasks can be achieved. Furthermore, NLP can enhance clinical workflows by continuously monitoring and providing advice to healthcare professionals concerning reporting. The implementation of various NLP techniques varies among applications. Tokenization is a common feature of all systems, and stemming is common in most systems. A segmentation step is crucial in many systems, with almost half incorporating this step. However, limited performance improvement has been observed in studies incorporating syntactic analysis [50–52]. Instead, systems frequently enhance their performance through the utilization of attributes originating from semantic analysis. This approach usually involves a specialized lexicon to detect relevant terms and their synonyms. These lexicons are typically crafted manually by experts in a particular field, but they can also be integrated with pre-existing lexicons [53–58].

The results of our study showed that to retrieve concepts from electronic texts recorded in the field of cancer, researchers have employed several methods and algorithms. The rule-based algorithm was the most frequently used algorithm in the included studies. However, deep learning has been used more frequently in healthcare [30, 59]; in certain studies that have compared rule-based and machine learning algorithms, it has been observed that both rule-based algorithms and machine learning classifiers can demonstrate comparable performance when evaluated using the same dataset [60, 61].In recent years, the popularity of machine learning algorithms has increased considerably, most likely due to their improved scalability and user-friendliness [62]. Despite the widespread adaption of deep learning methods, this study showed that both rule-based and traditional algorithms are still popular. A likely reason for this may be that these algorithms are simple and easier to implement and understand, as well as more interpretable compared to deep learning methods [63]. Interpretation of deep learning can be challenging because the steps that are taken to arrive at the final analytical output are not always as clear as those used in more traditional methods [63–65]. In addition, rule-based and traditional algorithms are more useful for smaller datasets with few features as these algorithms do not require massive amounts of data that are necessary for the development and successful implementation of machine learning. Furthermore, ML techniques can lead to a phenomenon known as overfitting, in which the developed model is too close to the underlying data set, which can limit the generalizability of the model to different data sets and making accurate predictions in other situations. However, this does not mean that using traditional algorithms is always a better approach than using deep learning since some situations may require more flexible and complex techniques [63].

Despite considerable variety among the evaluation methods when using NLP algorithms that have been reported in previous studies and published articles [66, 67], most of the retrieved articles in our study used the recall (R), f1-score (F1), and precision (P) metrics, to evaluate the findings of the algorithms being investigated. The recall ranged from 0.71 to 1.0, the precision ranged from 0.75 to 1.0, and the f1-score ranged from 0.79 to 0.93. The present study included articles that used pre-developed software or software developed by researchers to interpret the text and extract the cancer concepts. Pons et al. [13] systematically reviewed articles that used image processing software to automatically encode radiology reports. Similar to our study, this review extracted concepts identified by included studies, the NLP methodology and tools used, and their application purpose and performance results.

The most commonly used terminologies in the articles were UMLS and SNOMED-CT, among which UMLS was utilized more frequently [30]. A study in 2020 showed that 42% of UMLS users were researchers, and 28% of terminology users were programmers and software developers. Both groups acknowledged that terminologies were used to find concepts in the texts and the relationship between terms [68]. In this study, the articles concerning the use of UMLS were divided into six categories, with more than half of the articles (about 78%) falling under the NLP category [68].

The use of SNOMED-CT terminology in implementations has increased in recent years, while its use in theoretical discussions has recently been reduced [69]. The results of our study also indicated the practical use of this terminology to retrieve concepts from medical texts or documents.

In 2013, a review paper [70] on the application of SNOMED-CT in 2001 and 2012 categorized the included articles into five groups: unknown, theoretical, development and design, implementation, and evaluation. In this review, the number of studies related to implementation was 44 out of 488 relevant articles, which was a small number compared to the total number of articles. However, in the study by Change et al. [69], 124 articles out of 622 addressed this topic, which shows the importance of this field and the attention it has received in recent years. Most of these articles focused on the classification or coding of free-text clinical notes/narratives and radiology reports.

Despite the importance of content coverage as a metric in the evaluation of terminological systems, most of the articles included in our review did not include this information in their results, and only five articles reported this information. The reason can be that the focus of the included studies has been more on the extraction of the concepts from the narrative and identification of the best algorithms rather than the evaluation of applied terminological systems. Usually, studies that have been conducted to evaluate terminological systems focused on their content coverage [71, 72].

### Implication

The results of this study will help researchers to identify the most common techniques used to process cancer-related texts. This study also identified the terminologies that were mainly used to retrieve the concepts concerning cancer. The findings of this study will assist software developers in identifying the most beneficial algorithms and terminologies to retrieve the concepts from narrative text.

### Strengths and limitations of the study

In this article, in addition to examining NLP algorithms, we also reviewed the coding systems used for identifying concepts. This study had some limitations. We only searched for articles that were related to cancer-specific concepts. Studies that used the NLP technique in the field of cancer but extracted tumor features, such as tumor size, color, and shape, were excluded from the study. In addition, articles that used the results of tests and clinical examinations to diagnose cancer were also excluded. Articles that used AI and ML methods were also excluded from the study. One of the other limitations of this study was that due to the insufficiency of information concerning datasets used in the included studies, it was impossible to categorize studies based on the public and non-public nature of the datasets. Our contact with the authors of the articles did not reach any specific results. We suggest that future studies consider these limitations.

## Conclusions

This systematic review was the first comprehensive evaluation of NLP algorithms applied to cancer concept extraction. Information extraction from narrative text and coding the concepts using NLP is a new field in biomedical, medical, and clinical fields. The results of this study showed UMLS and SNOMED-CT systems are the most used terminologies in the field of NLP for extracting cancer concepts. We have also reviewed NLP algorithms that help researchers retrieve cancer concepts and found that rule-based methods were the most frequently used techniques in this field. Considering that limited studies applied ML and deep learning algorithms to extract concepts from the narrative text, it is recommended that researchers focus on the application of these methods in information extraction and synthesize the results of these types of studies. In addition, in the future, researchers can compare the results of natural language processing software to extract the concepts of various diseases from clinical documents such as radiology or laboratory reports. Moreover, as most of the included studies had not reported the content coverage of the applied terminological systems, future studies should address this type of results as it can help developers of the systems to choose the right terminological system with proper coverage.

## Data Availability

The datasets used and/or analyzed during the current study are available from the corresponding author upon reasonable request.

## Abbreviations

NLP: Natural language processing
M: Machine learning
